# Using Real-World Data to Study Deprescribing of Potentially Inappropriate Medications

**DOI:** 10.1093/geroni/igaf122.355

**Published:** 2025-12-31

**Authors:** Joshua Lin

## Abstract

Deprescribing potentially inappropriate medications (PIM) in a timely fashion is pivotal for drug safety in older adults. However, high-quality evidence to inform how to deprescribe PIM is often lacking. Randomized controlled trials (RCTs) typically severely underrepresent complex and frail patients in routine care. Insurance claims data and electronic health records (EHRs) are the two most commonly used real-world data sources in deprescribing research. Using 5 highly relevant examples, we will introduce common biases and mitigation strategies when using EHR and claims data for deprescribing research. Specifically, Study 1 integrated EHR with Medicare claims data to establish a scalable natural language processing (NLP)-based framework to validate claims-based medication discontinuation definitions. Study 2 demonstrated how one could use the Veterans Affairs nursing home EHR to study deprescribing of anticholinergic medications in which misclassification due to over-the-counter anticholinergic agent use can be minimized. Study 3 illustrated how we can investigate the rates, predictors, and barriers of deprescribing antipsychotics used for behavioral and psychological symptoms in dementia using a US national commercial insurance database (Optum’s Clinformatics DataMart [CDM]). The last two studies utilized both Medicare data and Optum CDM. Study 4 compared discontinuation vs. continuation of antipsychotics following hospitalization in older adults. Study 5 compared the likelihood of discontinuing chronic opioid therapy after initiating gabapentinoids vs. muscle relaxants among community-dwelling older adults with chronic back pain. With a variety of data sources and deprescribing clinical questions, we will discuss the opportunities and potential pitfalls of conducting deprescribing research using observational routine care data.

